# Energy-Efficient Unmanned Aerial Vehicle-Aided Visible Light Communication with an Angle Diversity Transmitter for Joint Emergency Illumination and Communication

**DOI:** 10.3390/s23187886

**Published:** 2023-09-14

**Authors:** Qiang Huang, Wanli Wen, Min Liu, Pengfei Du, Chen Chen

**Affiliations:** 1 School of Microelectronics and Communication Engineering, Chongqing University, Chongqing 400044, China; 202112021026@cqu.edu.cn (Q.H.); liumin@cqu.edu.cn (M.L.); c.chen@cqu.edu.cn (C.C.); 2 Singapore Institute of Manufacturing Technology (SIMTech), Agency for Science, Technology and Research (A*STAR), Singapore 636732, Singapore; du_pengfei@simtech.a-star.edu.sg

**Keywords:** visible light communication (VLC), unmanned aerial vehicle (UAV), angle diversity transmitter (ADT)

## Abstract

Unmanned aerial vehicle-aided visible light communication (UAV-VLC) can be used to realize joint emergency illumination and communication, but the endurance of UAV is a key limiting factor. In order to overcome this limitation, this paper proposes the use of an angle diversity transmitter (ADT) to enhance the energy efficiency of the UAV-VLC system. The ADT is designed with one bottom LED and several evenly distributed inclined side LEDs. By jointly optimizing the inclination angle of the side LEDs in the ADT and the height of the hovering UAV, the study aims to minimize the power consumption of the UAV-VLC system while satisfying the requirements of both illumination and communication. Simulation results show that the energy efficiency of the UAV-VLC system can be greatly enhanced by applying the optimized ADT. Moreover, the energy efficiency enhancement is much more significant when the LEDs in the ADT have a smaller divergence angle, or more side LEDs are configured in the ADT. More specifically, a 50.9% energy efficiency improvement can be achieved by using the optimized ADT in comparison to the conventional non-angle diversity transmitter (NADT).

## 1. Introduction

Visible light communication (VLC) has the advantages of sufficient spectrum resources, no electromagnetic interference radiation and high security [1,2,3], which can use light emitting diodes (LEDs) for signal transmission [4]. Therefore, VLC has attracted widespread attention as it can not only meet the communication needs, but also provide illumination. In recent years, VLC has been considered for many emerging applications such as underwater communication [5,6], the Internet of things [7], wireless human–machine interactions [8], infrastructure-to-vehicle communication [9] and emergency communication [10]. Particularly, unmanned aerial vehicle-aided VLC (UAV-VLC) has been considered as promising candidate for joint emergency illumination and communication [11,12,13].

In the outdoor environment, the LEDs providing illumination are usually fixed to the UAV [14]. These LEDs can send signals to users as transmitters, which makes it possible to establish a UAV-VLC system. Due to the UAV’s flexible mobile position and adaptive height [15], UAV-VLC systems are particularly suitable for nighttime searches, disaster rescue and other scenarios that require emergency illumination and communication at the same time [16]. However, the applicability of UAV-VLC systems is limited due to their endurance abilities being constrained by limited energy [17]. There are many existing technologies for improving the endurance of UAVs. In [18], a UAV was equipped with an LED transmitter and a joint optimization problem of power allocation and the UAV’s position was formulated under the constraints of power allocation, users’ service quality and the UAV’s position, aiming to maximize the sum rate of all users. In [19], the authors optimized the UAVs’ deployment, users’ association and power efficiency to meet users’ emergency illumination and communication needs. The authors of [20] provided a feasible and reliable scheme for UAVs to find the optimal trajectory and monitor coverage control. These schemes of combining VLC with UAVs using LEDs provide flexible emergency illumination and communication solutions for various application scenarios. However, all these previous studies have used a simple LED transmitter to provide both illumination and communication, while more advanced transmitter designs have not yet been considered in UAV-VLC systems.

Using an appropriate diversity combination scheme through an angle diversity receiver (ADR) at the receiver can improve the communication rate and system performance of users in VLC systems [21,22,23]. Moreover, an angle diversity transmitter (ADT) at the transmitter side can also enhance the performance of VLC systems [24,25]. Nevertheless, the use of ADTs in UAV-VLC systems has not yet been reported in the literature, and whether using an ADT can enhance the performance of a UAV-VLC systems is still an open question.

This paper aims to apply ADTs in UAV-VLC systems and further minimize the system power consumption by jointly optimizing the hovering height of the UAV and the inclination angle of the side LEDs in the ADT, so as to enhance the energy efficiency of the system while satisfying the requirements of both illumination and communication. The obtained simulation results demonstrate the feasibility and superiority of applying optimized ADT to substantially improve the energy efficiency of the UAV-VLC system. It is shown that the use of an optimized ADT can achieve a substantial 50.9% energy efficiency improvement compared to the use of a conventional non-angle diversity transmitter (NADT) in the considered UAV-VLC system.

## 2. System Model

**Figure 1:**
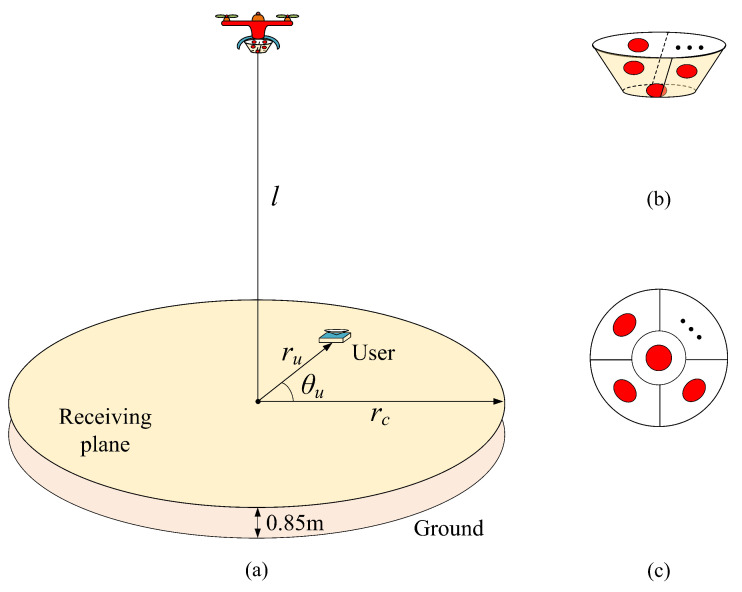
(**a**) System model of UAV-VLC using ADT, (**b**) geometric structure of ADT, and (**c**) bottom view of ADT.

In this section, we consider a general UAV-VLC system where the UAV is equipped with an ADT to provide simultaneous illumination and communication for a target area. The basic model of the UAV-VLC system using ADT is illustrated in Figure 1a, where the height of the UAV from the receiving plane is *l* and the radius of the UAV’s circular coverage area is $r_{c}$. By using the polar coordinate system, the location of a specific user over the receiving plane can be determined by its corresponding horizontal distance $r_{u}$ and polar angle $\theta_{u}$ [26]. Moreover, the ADT designed for the UAV-VLC system consists of totally *N* LEDs, including one bottom LED and N−1 inclined side LEDs. The geometric structure and the bottom view of the ADT are depicted in Figure 1b,c, respectively. In the ADT-assisted UAV-VLC system, all LEDs in the ADT are assumed to transmit the same electrical signal *x*, and hence the received electrical signal of the user located at position ($r_{u}$, $\theta_{u}$) can be expressed by

(1) $$y\left(t\right)=\mu \xi \sum_{i=1}^{N}h_{i}x\left(t\right)+\delta \left(t\right),$$

where μ is the photoelectric conversion coefficient of the LED, ξ is the responsivity of the photo-detector (PD) adopted by the user, $h_{i}$ is the optical channel gain corresponding to the *i*-th LED in the ADT with i=0,1,⋯,N−1, and δ(t) is the additive noise.

### 2.1. Principles of the ADT

**Figure 2:**
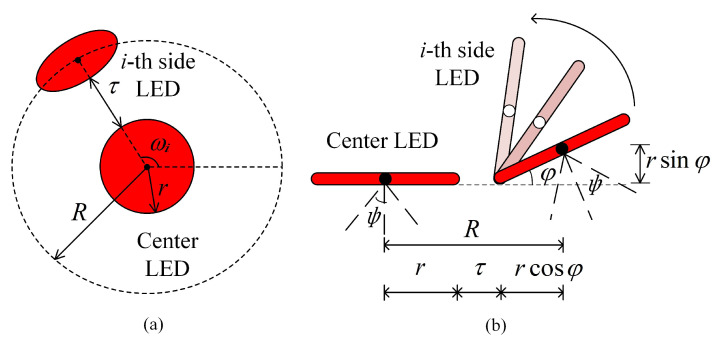
(**a**) Bottom view and (**b**) side view of the designed ADT. Only the bottom LED and the *i*-th side LED are displayed.

As shown in Figure 1b,c, the designed ADT consists of one bottom LED and N−1 inclined side LEDs. Here, we assume that all the LEDs in the ADT have the same optical and electrical performance. The bottom and side views of the designed ADT are depicted in Figure 2a,b, respectively, where only the bottom LED and the *i*-th side LED are shown for illustration. It can be seen that the bottom LED is located at the center of a circle with radius *R*, while the side LEDs are placed on the circumference of the circle and all the side LEDs have the same inclination angle φ. The circular LEDs have the same radius *r* and the gap between the bottom LED and the side LEDs is τ. In the ADT, the N−1 inclined side LEDs are evenly distributed on the circumference of the circle and the azimuth angle of the *i*-th side LED (i=1,⋯,N−1) is denoted as $\omega_{i}$. Assuming the azimuth angle of the first side LED is fixed at $\omega_{1}=0^{\circ }$, the azimuth angle of the *i*-th LED can be obtained by

(2) $$\omega_{i}=360^{\circ }\times \frac{i-1}{N-1},i=1,\ldots ,N-1.$$

**Figure 3:**
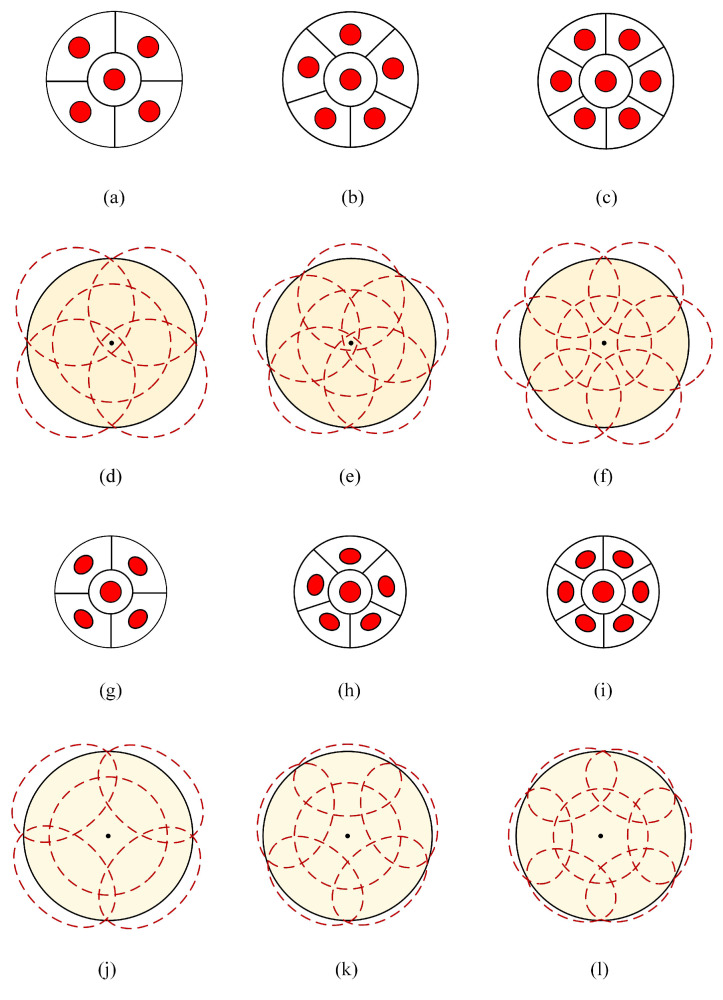
Bottom view of NADT for (**a**) *N* = 5, (**b**) *N* = 6, (**c**) *N* = 7, projection distribution at the receiving plane of NADT for (**d**) *N* = 5, *l* = 2.3 m, $\psi =45^{\circ }$, (**e**) *N* = 6, *l* = 2 m, $\psi =45^{\circ }$, (**f**) *N* = 7, *l* = 1.6 m, $\psi =45^{\circ }$, bottom view of ADT for (**g**) *N* = 5, (**h**) *N* = 6, (**i**) *N* = 7, and projection distribution at the receiving plane of ADT for (**j**) *N* = 5, *l* = 2.3 m, $\psi =35^{\circ }$, $\varphi =34^{\circ }$, (**k**) *N* = 6, *l* = 2 m, $\psi =35^{\circ }$, $\varphi =36^{\circ }$, (**l**) *N* = 7, *l* = 1.6 m, $\psi =35^{\circ }$, $\varphi =48^{\circ }$. Ellipses are used to approximate the actual projections for the purpose of conceptual illustration.

Figure 3 illustrates the bottom view and the corresponding projection distribution of the non-angle diversity transmitter (NADT) and ADT on the receiving plane for different cases. The key difference between NADT and ADT is that ADT has inclined side LEDs, while all the LEDs in NADT are vertically oriented towards to receiving plane. As can be seen, when the target area is fixed and full coverage is guaranteed, the use of an ADT can ensure that the light is more concentrated within the coverage area when compared with the use of NADT. Furthermore, the required semi-angle at half power of the LEDs in the ADT can be reduced when there are more side LEDs in the ADT, which can not only make the light distribution more uniform on the receiving plane, but also achieve a better communication rate for users and more fair communication services in the target area.

### 2.2. Optical Channel Gain

In typical outdoor environments, it is reasonable to assume that the user’s PD only receives the line-of-sight (LOS) optical signals from the corresponding LEDs in the ADT, since the non-line-of-sight (NLOS) components can be negligible due to the lack of reflecting surfaces [27]. For simplicity and without loss of generality, we assume that the UAV is in a horizontal hovering state and hence the equipped ADT is vertically oriented towards the receiving plane. In consequence, the bottom LED of the ADT is also vertically oriented, while the side LEDs are not vertically oriented. Moreover, the user’s PD is further assumed to be vertically oriented towards the sky.

#### 2.2.1. Bottom LED

Assuming each LED follows a generalized Lambertian radiation pattern, the optical channel gain between the bottom LED of the ADT and the PD of the user at the *j*-th position can be calculated by [28]

(3) $$h_{0j}=\frac{(m+1)A}{2\pi d_{0j}^{2}}\cos^{m}\left(\theta_{0j}\right)g_{f}g_{c}\cos\left(\gamma_{0j}\right),j\in U,$$

where m=−ln2/ln(cosψ) is the order of Lambertian emission with ψ being the semi-angle at half power of each LED, *A* is the active area of the PD, $d_{0j}=\sqrt{r_{u}^{2}+l^{2}}$ is the distance between the bottom LED of the ADT and the PD of the user at the *j*-th position, $\theta_{0j}$ and $\gamma_{0j}$ are, respectively, the emission angle and the incidence angle, $g_{f}$ is the gain of the filter, $g_{c}=\frac{n^{2}}{\sin^{2}\Phi }$ is the gain of the optical concentrator with *n* and Φ, respectively, denoting the refractive index and the half-angle field-of-view (FOV) of the optical concentrator, and *U* denotes a set of all possible user positions within the system coverage. It should be noted that $h_{0j}$ becomes zero if $\theta_{0j}\geq \psi$ or $\gamma_{0j}\geq \Phi$.

#### 2.2.2. Side LEDs

Because the side LEDs of the ADT are not vertically oriented towards the receiving plane, the emission angle of each side LED should be specified so as to calculate the corresponding LOS optical channel gain. Letting $\left({\hat{x}}_{0},{\hat{y}}_{0},{\hat{z}}_{0}\right)$ and $\left({\hat{x}}_{i},{\hat{y}}_{i},{\hat{z}}_{i}\right)$, respectively, denote the coordinates of the bottom center LED and the *i*-th side LEDs, after some geometric calculations, we can obtain the following relationship:

(4) $$\left\{\begin{array}{c} {\hat{x}}_{i}={\hat{x}}_{0}+\left[\tau +r\left(1+\cos\varphi \right)\right]\cos\omega_{i}\\ {\hat{y}}_{i}={\hat{y}}_{0}+\left[\tau +r\left(1+\cos\varphi \right)\right]\sin\omega_{i}\\ {\hat{z}}_{i}={\hat{z}}_{i}+r\sin\varphi \end{array}\right..$$

**Figure 4:**
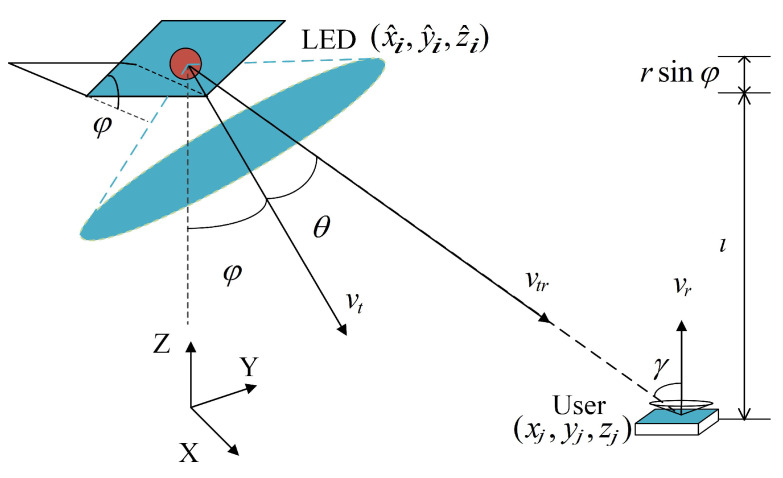
Geometry for LOS channel gain calculation of the side LEDs.

Figure 4 depicts the geometric structure used to calculate the optical channel gain of the side LEDs. The angle of incidence can be determined by the positions of the LEDs and the users. Let $\left(x_{j},y_{j},z_{j}\right)$ denote the coordinates of the user position; according to Figure 1a, we have

(5) $$\cos\phi_{ij}=\frac{{\hat{z}}_{i}-z_{j}}{{\left[{\left({\hat{x}}_{i}-x_{j}\right)}^{2}+{\left({\hat{y}}_{i}-y_{j}\right)}^{2}+{\left({\hat{z}}_{i}-z_{j}\right)}^{2}\right]}^{1/2}}.$$

However, the emission angle depends not only on the positions of the LEDs and the users, but also on the LED’s inclination angle φ and azimuth angle Ω. The azimuth angle of *i*-th side LED can be expressed as:

(6) $$\Omega_{i}=\omega_{i}+\beta ,$$

where $\omega_{i}$ is the azimuth angle of the *i*-th LED when the UAV is not rotated which is defined in (2), and β is the rotation angle of the UAV which is assumed to follow an uniform distribution between $0^{\circ }$ and $360^{\circ }$. Let $v_{i}$ and $v_{ij}$, respectively, denote the normal vector of the *i*-th side LED’s plane and the vector from the *i*-th side LED to the user at the *j*-th position, we have

(7) $$\cos\theta_{ij}=\frac{\left(v_{i},v_{ij}\right)}{∥v_{i}∥\cdot ∥v_{ij}∥},$$

where $\left(\cdot ,\cdot \right)$ and $∥\cdot ∥$ represent the inner product of two vectors and the norm of a vector, respectively. For the *i*-th side LED with an azimuth angle $\Omega_{i}$ and an inclination angle φ, the normal vector of the *i*-th side LED’s plane is given by

(8) $$v_{i}=\left(\sin\varphi \cos\Omega_{i},\sin\varphi \sin\Omega_{i},-\cos\varphi \right).$$

Moreover, the vector from the *i*-th side LED to the user at the *j*-th position is also given by

(9) $$v_{ij}=\left(x_{j}-{\hat{x}}_{i},y_{j}-{\hat{y}}_{i},z_{j}-{\hat{z}}_{i}\right).$$

Substituting (8) and (9) into (7), we have

(10) $$\cos\theta_{ij}=\frac{\left[\left(x_{j}-{\hat{x}}_{i}\right)\cos\Omega_{i}+\left(y_{j}-{\hat{y}}_{i}\right)\sin\Omega_{i}\right]\sin\varphi -\left(z_{j}-{\hat{z}}_{i}\right)\cos\varphi }{{\left[{\left(x_{j}-{\hat{x}}_{i}\right)}^{2}+{\left(y_{j}-{\hat{y}}_{i}\right)}^{2}+{\left(z_{j}-{\hat{z}}_{i}\right)}^{2}\right]}^{1/2}}.$$

Finally, the LOS optical channel gain between the *i*-th LED in the ADT and the PD of the user at the *j*-th position can be calculated by

(11) $$h_{ij}=\frac{(m+1)A}{2\pi d_{ij}^{2}}\cos^{m}\left(\theta_{ij}\right)g_{f}g_{c}\cos\left(\gamma_{ij}\right),$$

where $d_{ij}={\left({\left({\hat{x}}_{i}-x_{j}\right)}^{2}+{\left({\hat{y}}_{i}-y_{j}\right)}^{2}+{\left({\hat{z}}_{i}-z_{j}\right)}^{2}\right)}^{1/2}$ is the distance between the *i*-th LED in the ADT and the *j*-th user and $\gamma_{ij}$ is the corresponding incident angle.

Since all the LEDs in the ADT transmit the same signal, the overall optical channel gain between the the ADT and the PD of the user at the *j*-th position is obtained by

(12) $$h_{j}=\sum_{i=0}^{N-1}h_{ij}.$$

### 2.3. Energy Efficiency

Due to the limited energy of a typical UAV, it is of practical significance to improve the energy efficiency of the UAV-aided VLC system while ensuring both emergency communication and illumination requirements. The objective of this study is to determine the optimal hovering height of the UAV and the optimal inclination angle of the side LEDs in the ADT so as to minimize the system power consumption. It should be noted that this study only focuses the power consumption of LED-based illumination and communication.

The achievable rate, i.e., the spectral efficiency for a given signal bandwidth, of the user at the *j*-th position can be approximated by [29]:

(13) $$C_{j}=\frac{1}{2}\log_{2}\left(1+\frac{e}{2\pi }{\left(\frac{\varepsilon P_{j}h_{j}}{\sigma_{w}}\right)}^{2}\right),$$

where ε is the illumination target [27], $P_{j}$ refers to the optical power of the UAV, and $\sigma_{w}$ represents the standard deviation of the additive white Gaussian noise.

Assuming the data rate threshold for the user to establish a successful communication link is $C_{\mathrm{th}}$, according to (13), the minimum optical power to meet the communication requirement at the *j*-th user position can be obtained by

(14) $$P_{j,min}^{Communication}=\frac{\sigma_{w}\sqrt{\frac{2\pi }{e}\left(2^{2C_{\mathrm{th}}}-1\right)}}{\varepsilon h_{j}},j\in U.$$

Moreover, the illuminance at the *j*-th user position can be calculated by [30]

(15) $$\kappa_{j}=\varepsilon P_{j}\sum_{i=0}^{N-1}\frac{(m+1)A}{2\pi d_{ij}^{2}}\cos^{m}\left(\theta_{ij}\right)g_{c}\cos\left(\gamma_{ij}\right).$$

Letting $\kappa_{th}$ denote the illuminance threshold for efficient illumination, according to (15), the minimum optical power to meet the illumination requirement at the *j*-th user position can be achieved by

(16) $$P_{j,min}^{Illumination}=\frac{\kappa_{th}}{\varepsilon \sum_{i=0}^{N-1}\frac{(m+1)A}{2\pi d_{ij}^{2}}\cos^{m}\left(\theta_{ij}\right)g_{c}\cos\left(\gamma_{ij}\right)},j\in U.$$

In order to meet the requirements of both communication and illumination for all the possible user positions, the minimum optical power can be obtained as follows:

(17) $$P_{min}=\max_{j\in U}\left\{P_{j,min}^{Communication},P_{j,min}^{Illumination}\right\},$$

which corresponds to the $j^{*}$-th user position.

Therefore, the corresponding energy efficiency of the UAV-VLC system can be obtained as follows:

(18) $$\eta =\frac{C_{j^{*}}}{P_{min}}=\frac{1}{2P_{min}}\log_{2}\left(1+\frac{e}{2\pi }{\left(\frac{\varepsilon P_{min}h_{j^{*}}}{\sigma_{w}}\right)}^{2}\right),$$

where $C_{j^{*}}$ denotes the achievable rate of the user at the $j^{*}$-th position.

## 3. Simulation Results

In this section, we evaluate the energy efficiency of the ADT-assisted UAV-VLC system in a practical deployment scenario for joint emergency illumination and communication, where multiple UAVs are deployed to cover the overall target area and a single UAV only covers a relatively small target area with a relatively low hovering height. More specifically, the radius of the circular target area of the UAV is assumed to be in the range from 1.4 m to 2.2 m with a step of 0.2 m, and the semi-angle at half power ψ of LEDs in the ADT are assumed to be $25^{\circ }$, $30^{\circ }$ and $35^{\circ }$. As a trade-off between performance and cost, the number of LEDs in the ADT is set to 5, 6 and 7. Moreover, the height of receiving plane from the ground is 0.85 m, the radius of LEDs in the ADT is 5 mm, and the gap between the center LED and the side LEDs is 5 cm. In order to support simultaneous emergency illumination and communication, the illuminance threshold and the channel capacity threshold are assumed to be $5\times 10^{-4}$ Lx and 1 bit/s/Hz, respectively, [18,19,27]. The other key simulation parameters can be found in Table 1.

Moreover, we compare the optimized ADT with NADT under different semi-angles at half the power of the LEDs in the following evaluations, and the reason can be explained as follows. Due to the inclination of side LEDs in the ADT, the overall coverage of the ADT is larger than that of the NADT, if the LEDs have the same semi-angle at half power. As a result, it is reasonable to reduce the semi-angle at half the power of the LEDs in the ADT as long as the ADT can successfully cover the target area. With the reduction in the semi-angle at half the power of the LEDs in the ADT, both the illumination and communication performance can be improved by using ADT in comparison to conventional NADT. Therefore, the comparison is made under the condition that the target area can be satisfactorily served by both ADT and NADT. More specifically, for a given semi-angle at half power of LEDs in the ADT, the semi-angle at half power of LEDs in the NADT is selected as the minimum integer value that can ensure the NADT to fully cover the target area.

**Figure 5:**
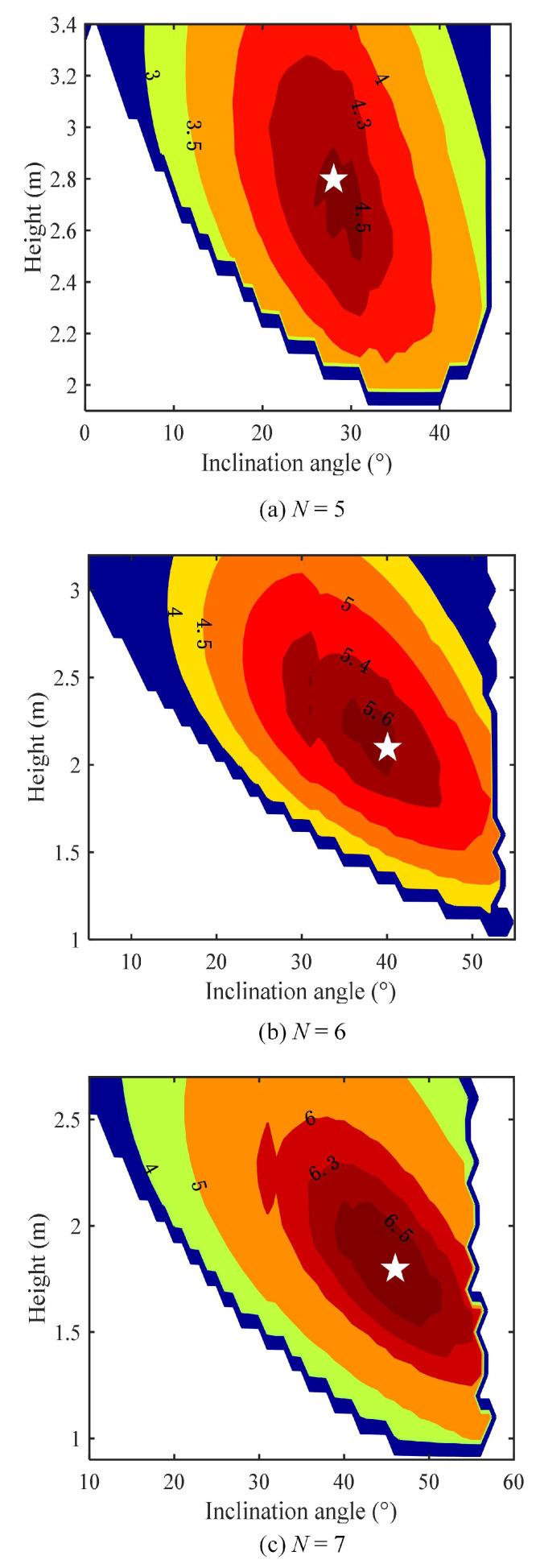
Contour plot of energy efficiency (bits/J/Hz) in terms of inclination angle φ and hovering height *l* with ψ = $30^{\circ }$ for (**a**) *N* = 5, (**b**) *N* = 6, and (**c**) *N* = 7. The white star denotes the optimal combination of inclination angle φ and hovering height *l* to achieve the maximum energy efficiency.

Figure 5 depicts the contour plot of the energy efficiency with unit bits/J/Hz in terms of the inclination angle φ of ADT and the hovering height *l* of UAV with ψ = $30^{\circ }$ for different *N* values, where the radius of the circular target area is set to 2 m. As we can see, there exists an optimal combination of inclination angle φ and hovering height *l* (highlighted by a white star) to achieve the maximum energy efficiency for given *N* and ψ, which verifies that it is feasible to optimize the performance of the ADT-assisted UAV-VLC system via jointly optimizing the inclination angle φ of ADT and the hovering height *l* of UAV. Note that the blank part in each contour plot indicates that the corresponding combination of inclination angle φ, and hovering height *l* cannot ensure the requirements of both emergence communication and illumination. Table 2 summarizes the obtained optimal $\varphi_{opt}$ and $l_{opt}$ and the corresponding maximum energy efficiency $\eta_{max}$ for different *N* and ψ values. It can be found from Table 2 that, with the increase in the semi-angle at half the power ψ of the LEDs in the ADT, the optimal inclination angle $\varphi_{opt}$ is gradually increased while the optimal hovering height $l_{opt}$ is gradually decreased, and the achievable maximum energy efficiency $\eta_{max}$ is reduced accordingly. Moreover, when the ADT consists of more LEDs, $\varphi_{opt}$ is increased, $l_{opt}$ is decreased, while $\eta_{max}$ can be improved.

**Figure 6:**
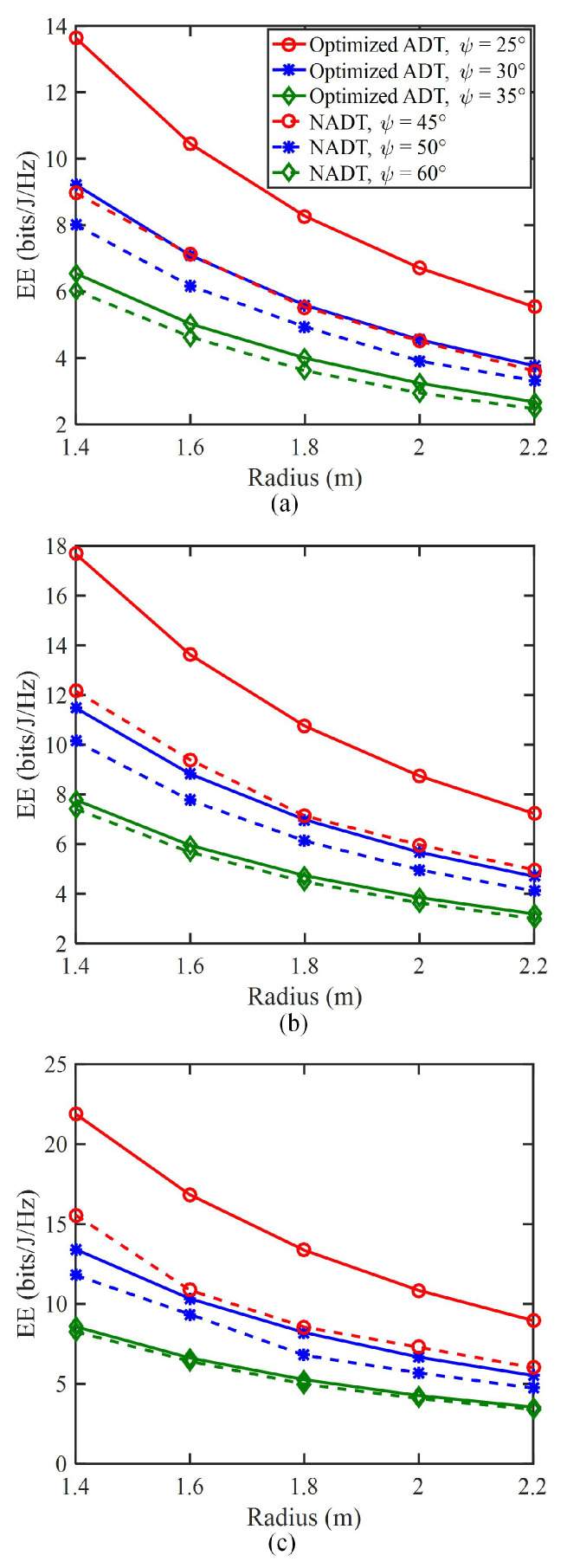
Energy efficiency (bits/J/Hz) vs. the radius of target area for (**a**) *N* = 5, (**b**) *N* = 6, and (**c**) *N* = 7.

Figure 6 shows the energy efficiency versus the radius of target area for different *N* values, where both the optimized ADT and the conventional NADT with different ψ values are considered for performance comparison. For the optimized ADT, three suitable ψ values, i.e., $25^{\circ }$, $30^{\circ }$ and $35^{\circ }$, are selected. To enable full coverage of the target area, the three ψ values for the conventional NADT are assumed to be $45^{\circ }$, $50^{\circ }$ and $60^{\circ }$. For *N* = 5, as shown in Figure 6a, the energy efficiency gradually decreases with the increase in the radius of target area for all the considered transmitter schemes, which indicates that the use of a small target area can efficiently enhance the energy efficiency of the UAV-VLC system. Moreover, for both the optimized ADT and the conventional NADT, a higher energy efficiency can be obtained when a relatively smaller semi-angle at half power ψ of the LEDs in the ADT is used. It can be further observed that the energy efficiency improvement from using the optimized ADT over the conventional NADT becomes much more significant when the semi-angle at half the power ψ of the LEDs in the ADT is relatively smaller. More specifically, for a target area radius of 1.8 m, the energy efficiencies for NADT with $\psi =45^{\circ }$ and optimized ADT with $\psi =25^{\circ }$ are, respectively, 5.5 and 8.3 bits/J/Hz, which corresponds to a substantial energy efficiency improvement of 50.9%. Furthermore, when there are more LEDs in the ADT, i.e., the *N* value becomes larger, the energy efficiency is greatly improved for all the considered transmitter schemes.

**Figure 7:**
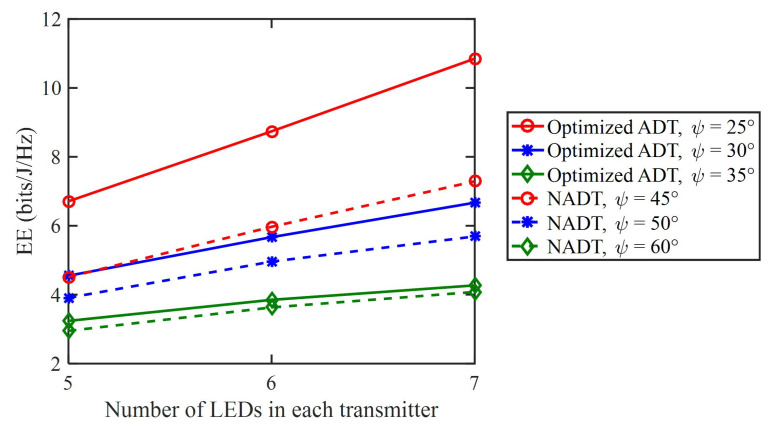
Energy efficiency vs. the number of LEDs in each transmitter.

Figure 7 depicts the relationship between the energy efficiency and the number of LEDs in each transmitter. As we can clearly see, all types of transmitters exhibit an increase in energy efficiency as the number of LEDs increases. Particularly, for the optimized ADT with $\psi =25^{\circ }$, the energy efficiency is improved from 6.7 to 10.9 bits/J/Hz, when *N* is increased from 5 to 7, which corresponds to a substantial energy efficiency improvement of 62.7%. Notably, the optimized ADT exhibits a much more significant improvement in energy efficiency compared to NADT, especially when the semi-angle at half power ψ of the LEDs in the ADT becomes smaller.

## 4. Conclusions

In this paper, we have proposed and evaluated an ADT-assisted UAV-VLC system for joint emergency illumination and communication, where the ADT consists of one bottom LED and several inclined side LEDs. By jointly optimizing the hovering height of the UAV and the inclination angle of the sides LEDs in the ADT, the power consumption of the ADT-assisted UAV-VLC system can be minimized, and hence the system energy efficiency can be enhanced, under the condition of satisfying the requirements of both illumination and communication. The presented simulation results have successfully verified the feasibility and superiority of applying optimized ADT to enable an energy-efficient UAV-VLC system. It is revealed that a 50.9% energy efficiency improvement can be achieved by using the optimized ADT in comparison to the conventional (NADT) and, meanwhile, the energy efficiency can be enhanced by 62.7% when the number of LEDs in the ADT is increased from 5 to 7. Therefore, the proposed optimized ADT-assisted UAV-VLC can be a promising candidate for practical joint emergency illumination and communication scenarios.

In this current work, only a single UAV is considered in the UAV-VLC system, which might not be sufficient to serve a large coverage area. In our future work, we will consider a practically distributed UAV-VLC system where multiple UAVs work together to serve a large coverage area.

## Figures and Tables

**Table 1:** Simulation Parameters.

Parameter	Value
Radius of target area	1.4 m, 1.6 m, 1.8 m, 2 m, 2.2 m
Height of receiving plane	0.85 m
Number of LEDs in ADT	5, 6, 7
Radius of LEDs in ADT	5 mm
Gap between center and side LEDs	5 cm
Semi-angle at half power of LEDs	$25^{\circ }$, $30^{\circ }$, $35^{\circ }$
Photoelectric conversion coefficient	1 W/A
Gain of filter	0.9
Half-angle FOV of optical concentrator	$65^{\circ }$
Refractive index of optical concentrator	1.5
Active area of PD	1 cm^2^
Noise PSD	$1\times 10^{-22}$ A^2^/Hz
Illuminance threshold	$5\times 10^{-4}$ Lx
Achievable rate threshold	1 bit/s/Hz
Responsivity	0.8 A/W
Signal bandwidth	10 MHz

**Table 2:** Optimization results.

*N*	ψ	$\varphi_{\mathrm{opt}}$	$l_{\mathrm{opt}}$	$\eta_{\max}$
	$25^{\circ }$	$24^{\circ }$	3.3 m	6.7 bits/J/Hz
*N* = 5	$30^{\circ }$	$28^{\circ }$	2.8 m	4.6 bits/J/Hz
	$35^{\circ }$	$34^{\circ }$	2.3 m	3.2 bits/J/Hz
	$25^{\circ }$	$32^{\circ }$	2.7 m	8.7 bits/J/Hz
*N* = 6	$30^{\circ }$	$40^{\circ }$	2.1 m	5.7 bits/J/Hz
	$35^{\circ }$	$36^{\circ }$	2 m	3.8 bits/J/Hz
	$25^{\circ }$	$39^{\circ }$	2.2 m	10.8 bits/J/Hz
*N* = 7	$30^{\circ }$	$46^{\circ }$	1.8 m	6.7 bits/J/Hz
	$35^{\circ }$	$48^{\circ }$	1.6 m	4.3 bits/J/Hz

## Data Availability

Not applicable.
